# Biomarkers and mechanisms of tolerance induction in food allergic patients drive new therapeutic approaches

**DOI:** 10.3389/fimmu.2022.972103

**Published:** 2022-10-03

**Authors:** Carolyn H. Baloh, Michelle F. Huffaker, Tanya Laidlaw

**Affiliations:** ^1^ Immune Tolerance Network, Benaroya Research Institute at Virginia Mason, Seattle, WA, United States; ^2^ Department of Medicine, Harvard Medical School, the Division of Allergy and Clinical Immunology, Brigham and Women’s Hospital, Boston, MA, United States; ^3^ Immune Tolerance Network, University of California San Francisco, San Francisco, CA, United States

**Keywords:** peanut allergy, food allergy, tolerance, allergen immunotherapy (AIT), oral immunotherapy (OIT)

## Abstract

Immunotherapy for food-allergic patients has been effective in inducing desensitization in some populations, but long-term tolerance has remained an elusive target. A challenge facing our field is how to differentiate immune markers that are impacted by immunotherapy from those that are critical biomarkers of tolerance. Data from recent clinical trials have identified several biomarkers and mechanisms for achieving tolerance. These biomarkers include younger age, lower food-specific IgE, lower food component-specific IgE, specific linear epitope profiles, and subsets of food-specific CD4+ T cells. Additional biomarkers under investigation for their relevance in tolerance induction include TCR repertoires, gastrointestinal and skin microbiome, and local tissue immunity. This mini-review highlights recent advances in understanding biomarkers and mechanisms of tolerance induction in food immunotherapy and how these are influencing clinical trial development.

## Introduction

Global estimates of the rate of food allergy are as high as 11%, with higher prevalence amongst children and in Western countries (1–3). Despite the frequency of food allergies, the most common clinical management strategy is to avoid the offending food and carry an epinephrine autoinjector. Multiple small trials of peanut oral immunotherapy (OIT) conducted from the 1990s to 2010s and assessed in a meta-analysis suggested that the threshold of reactivity to peanut could be improved through OIT (4). The landmark Peanut Allergy Oral Immunotherapy Study of AR101 for Desensitization in Children and Adults (PALISADE) trial, a phase 3 trial of OIT with AR101, a peanut-derived powder, provided the confirmatory evidence in a large clinical trial that we could modulate the immune system of a large population of peanut-allergic patients and prevent their reactions to peanut consumption (5). In 2020, the FDA approved the first OIT product for peanut allergy based on this trial (6). Importantly, the primary outcome of the PALISADE trial was desensitization, defined as the ability to consume a food without allergic reaction while still on therapy. Subsequent trials have aimed to achieve tolerance, which is defined as the ability to consume a food without reaction even after stopping therapy. Numerous terms synonymous with tolerance are used in the literature including sustained unresponsiveness and remission.

Achievement of tolerance has proven to be relatively elusive, leading to the investigation of new approaches including altering the route of immunotherapy (e.g. sublingual, epicutaneous), adding a biologic or adjuvant to immunotherapy, vaccines, and peptide therapy. Mechanistic studies have been and are being performed in association with clinical trials to elucidate the immune mechanisms necessary to achieve desensitization and subsequent tolerance. Improving our understanding of these mechanisms is essential to moving this field forward. The aim of this mini-review is to discuss known mechanisms to achieve desensitization and/or tolerance with an eye on how this can inform design of future clinical trials.

## Immunologic response to immunotherapy

### Innate immune response

Examination of the innate immune response to immunotherapy (IT) has revealed novel mechanisms of desensitization and tolerance. Dendritic cells play an important role in the pathophysiology of atopy through antigen presentation, pro-inflammatory cytokine production, and preferential promotion of Th2 differentiation over Treg differentiation (7). Peanut OIT, SLIT, and milk SLIT all decrease TLR-induced pro-inflammatory cytokine production of monocytic dendritic cells (8, 9). Peanut OIT has also been shown to lead to a partial improvement in impaired IFN-α secretion by plasmacytoid dendritic cells (7, 8, 10). It is likely that dendritic cells have roles in tolerance, but replication of the above findings and additional clarification of pro-tolerogenic roles is needed.

### Cytokine response

Atopy, and food allergy specifically, is known to be mediated primarily by the Th2 axis of the adaptive immune system. Most early studies of food immunotherapy have shown that Th2 cytokines are decreased by OIT. Specific cytokines for which food immunotherapy causes decreases in peripheral blood levels include IL-5 (11–14), IL-4 (13), and IL-13 (12, 14). However, IL-5 levels have also been shown to increase in response to peanut OIT (15).

While mouse studies have shown that either maintenance of adequate levels or increased levels of Th1 cytokines and the regulatory cytokine IL-10 in OIT may be important for tolerance development, there are few studies looking at this in humans (16, 17). One trial of peanut SLIT found no changes in IL-10 or IFN-γ in participants who received SLIT (11). Some trials of peanut OIT found a reduction in IL-10 (13, 14), IFN-γ (14), and TNF-α (14), but one trial of peanut OIT had conflicting findings of an increase in IL-10, IFN-γ, and TNF-α (15). The role of these cytokines in tolerance requires further study and is becoming increasingly relevant in an era of targeted biologics.

### Humoral immune response

It is well-established that food-specific IgE levels increase transiently early in treatment with food IT but are thereafter decreased (11, 18–21). IgE to food-specific components are also decreased in response to immunotherapy (21–24). While lower baseline food-specific IgE has been identified as a biomarker of tolerance development (discussed below), no trials have found that decreasing food or component-specific IgE below a particular threshold led to tolerance.

Food and component-specific IgG4 are increased by food IT (11, 12, 18, 20, 22, 23), but again, neither changes in IgG4 levels nor the ratio of food-specific IgG4 to food-specific IgE have been associated with tolerance development. It is also possible that IgG4 doesn’t correlate with tolerance because it is a reflection of exposure to the allergen prior to IT initiation and is thus reflective of less severe allergy at baseline. Food immunotherapy trials have largely not measured other IgG subclasses (25), though *in vitro* studies suggest that IgG2 & IgG3 are also relevant to blocking mast cell degranulation (26, 27). Additional studies are needed to measure IgG1, 2, 3, and 4 levels and relate them to food immunotherapy outcomes.

Immunotherapy has also been shown to alter IgE binding to sequential (linear) peanut epitopes. In a trial of milk OIT, significant changes were induced in 73% of IgE-binding and 91% of IgG4-binding epitopes and epitope diversity was significantly decreased for IgE but not IgG4-specific epitopes (28). Overall, OIT-induced changes in epitope diversity were not different between participants who achieved desensitization without tolerance compared to those who achieved desensitization and tolerance (28).

Studies of B cells in food IT are limited in part due to their low frequency in peripheral blood (29). One study using tetramer-based approach found that peanut OIT led to an early increase in affinity matured, somatically hypermutated, oligoclonal Ara h 2 specific memory B cells (30). Another study utilized sorting of Ara h 1 or 2 reactive B cells followed by deep sequencing of the B cell repertoire and discovered that immunotherapy induces somatic mutations in IgG4 (29). A potential implication of this finding is that OIT may induce IgG4 with a higher affinity to block IgE-mediated mast cell degranulation. With recent technological advances, including single cell omics and use of tetramers, we may be better able to understand the role of B cells in response to immunotherapy.

### T cell response

Antigen-specific Th2 CD4+ cells most critical to food allergy pathogenesis and induction of tolerance are defined differently across studies and it appears that there is heterogeneity within this population of cells (21, 31–33). Regardless of whether these cells are defined as IL-4 and/or IL-13 producing antigen-specific CD4+ cells, as antigen-specific Th2A cells (CD4^+^CD45RO^+^CD27^-^CD45RB^lo^CRTH2^+^CD49d^+^CD161^+^), or by their transcriptomic signature, they exhibit a sharp decrease early (by 12 weeks) in IT and a continued slower decrease thereafter (20, 21, 33). These cells also drop to lower frequencies in patients who go on to develop desensitization (32), implying that depletion of this cell population may be a crucial step in the pathway to tolerance. However, whether deletion, anergy, or exhaustion is required is still under investigation. There are two studies to date that suggest development of an anergic cluster of T cells is necessary for desensitization to food allergens (33, 34), but studies in mice and/or of aeroallergen desensitization in humans provide mixed data about whether deletion, anergy, or exhaustion is required (31, 35–38). One study of peanut OIT also suggests that transient changes in antigen-specific Th2 CD4+ cells may also be relevant for tolerance (39).

Conceptually, it would make sense that Treg cells are important in tolerance development. However, data from immunotherapy (IT) studies on Treg frequency differ with some studies finding that IT leads to increases in Tregs (12, 40), others finding the opposite (21), and still others finding no impact of IT on Tregs (11, 21). This may be in part due to differences in how Tregs are defined or which subtypes are evaluated in each study.

A recently identified subset of T follicular helper cells (Tfh), Tfh13 cells, has been shown to have a key role in the pathogenesis of food allergy through production of high-affinity IgE (41). Unfortunately, evidence is again conflicting as to whether these cell populations are altered by IT and whether modulation of these cells is needed for tolerance (33, 39).

### Basophil response

The basophil activation test (BAT) is a laboratory assay whereby a participant’s basophils are stimulated with the relevant allergen and measures of basophil activation (reactivity) are recorded (42). BAT is useful in the diagnosis of food allergy and is often measured in clinical trials of food IT (9, 14, 20, 22, 40, 42–46). It has been well-established that oral immunotherapy (OIT), sublingual immunotherapy (SLIT), and epicutaneous immunotherapy (EPIT) all result in decreases in BAT (14, 20, 43, 45). Lower basophil reactivity can be seen as early as the build-up phase of immunotherapy and either continues to decrease or remains stable during maintenance (22, 43, 45). Several studies, including studies of peanut OIT and milk OIT/SLIT have found that early decreases in BAT, during or at the end of immunotherapy build-up, predict later development of tolerance (43, 44). In the Peanut Oral Immunotherapy Study: Safety, Efficacy and Discovery (POISED) trial, lower basophil activation throughout the trial correlated with development of tolerance (45). Notably, decreases in basophil reactivity have been identified to be transient, often rebounding after immunotherapy is discontinued (9, 22, 45). Despite the rebound, participants who achieved clinical tolerance in POISED and another trial, the Peanut Oral Immunotherapy in Children (IMPACT) trial, had less increase in basophil reactivity after discontinuation of IT than did participants who did not achieve tolerance (22, 45). Together, these data suggest that decreasing basophil reactivity is necessary to achieve clinical desensitization and tolerance. It is possible that we achieve low rates of tolerance in food immunotherapy clinical trials in part because we have not yet achieved a sufficient degree of basophil blocking and/or suppression or the mechanism by which we accomplish basophil blocking and/or suppression is not permanent.

## Baseline biomarkers that predict desensitization and tolerance

In addition to understanding mechanisms by which tolerance can be achieved, there is utility in recognizing biomarkers that are predictive of tolerance prior to initiation of immunotherapy. Baseline biomarkers identified in clinical trials include food-specific antibodies, basophil activation tests, and T cell subsets. Clarification of such biomarkers would provide clinicians with tools to select and appropriately counsel patients and their families regarding likelihood of achieving a good clinical response to immunotherapy. Clinical trials targeting biomarkers of poor response with specific adjuvants to immunotherapy may be a worthwhile approach to improve tolerance outcomes.

### Age as a predictor of desensitization or tolerance

Age has garnered much discussion in relation to food immunotherapy outcomes. The Learning Early About Peanut Allergy (LEAP) study, a primary prevention study of peanut allergy in high-risk infants, provided the first evidence that peanut allergy can be largely prevented through early peanut introduction (47). Multiple peanut OIT studies have shown that tolerance can best be induced in patients with known peanut allergy at younger ages (22, 48), however, trials in older children did not find age impacted outcomes (24, 49). This suggests an early immune malleability that disappears with age.

### Specific IgE as a predictor of desensitization or tolerance

The most well-studied mechanistic biomarker of tolerance has been food-specific IgE, and multiple trials of milk, egg, and peanut OIT have shown that lower baseline IgE correlates with better outcomes (18, 22, 24, 48, 50, 51) with rare exception (21). Other immune globulins or immune globulin components likely play a role as well. For example, in a trial of peanut OIT, a lower total IgE and a higher peanut-specific IgG4:IgE ratio independently associated with higher rates of tolerance (24). Lower IgE to food components including ovalbumin, casein, and Ara h 1, 2, 3 and 6 have also been identified as potential biomarkers of desensitization and tolerance with varying significance (18, 21–24, 50, 51).

IgE binding to sequential (linear) epitopes has been shown to have a high diagnostic accuracy for diagnosis of peanut, milk, and egg allergy (52–54). Further, the pattern of IgE-binding epitopes identified at baseline for a cohort of participants undergoing milk OIT differentiated those who achieved desensitization only, desensitization and tolerance, or failed therapy (55).

### Basophil activation as a predictor of desensitization or tolerance

While BAT has demonstrated utility for predicting outcomes during OIT, data regarding whether BAT prior to initiation of immunotherapy is a useful biomarker of subsequent tolerance are mixed, possibly due to variation in specimen type, processing, or assay protocol (9, 21, 40, 42, 43, 45, 46). Standardization is needed before we can determine the clinical utility of baseline BAT in predicting tolerance outcomes.

### T cell subsets as predictors of desensitization or tolerance

Interrogation of Th2 cell subsets has led to correlation of specific subsets with atopy (32) and ability to achieve tolerance in OIT trials (21). In CoFAR7, a lower number of peanut-specific T cells expressing IL-4 or IL-13 separated those who were able to achieve desensitization or tolerance from treatment failures. Other baseline assessments including peanut-specific T cells expressing IL-10 or IFN-γ did not correlate with clinical outcomes (21). Alternatively, Monian et al. found that expression of Th2 gene signature was not associated with tolerance, but that lower expression of gene module defined by T cell activation and effector response (OX40, OX40L, Th17 function, STAT1, and GPR15), lower Th1-conv, and lower Th17 cells were associated with tolerance (33). Limited data have been published on Tregs as predictors of OIT outcome. A study of egg OIT, baseline Treg frequency did not correlate with outcomes (21). Additional studies are needed to determine whether it is the quantity of or function of T cell subsets at baseline that relate to achieving tolerance.

## Newer biomarkers/mechanisms of tolerance under investigation

In addition to continuing investigation of the mechanisms of immunotherapy and biomarkers of tolerance discussed above, knowledge and technological advances are allowing us to expand research into other potential mechanisms of tolerance. These include TCR repertoires, the microbiome, and the role of the epithelial barrier.

The TCR repertoire can now be reliably measured after antigen stimulation to isolate food antigen-specific T cells (56, 57). In one study of 27 peanut-allergic individuals, there were TCRβ sequences shared across individuals suggesting that these sequences could be important in the pathogenesis of peanut allergy and specific epitope recognition (57). A follow-up study examined the TCR repertoire in response to peanut OIT and found that the repertoire was not dramatically impacted by OIT (33).

The microbiome from the skin and gut are skewed in individuals with food allergy. Specific gut microbes have been correlated with the development of food allergy and recent studies have suggested that these microbes relate to alterations in the metabolome and subsequent immune deviation (58–65). This is also an area that could be a potential therapeutic target, as peanut OIT may expand the diversity of the gut microbiome (66).

The gut epithelial barrier is also known to play a role in the pathogenesis of food allergy, largely through antigen uptake and cytokine production (TSLP, IL-33, and IL-15). The gut epithelium is also home to a many types of immune cells that are postulated to be relevant to food allergy development and tolerance induction. To date, only one clinical trial has examined gut epithelial biopsies during food OIT and they identified that tissue eosinophilia often develops during OIT but is usually transient (67).

## How biomarkers influence future clinical trial and therapeutic drug development

In the current era of targeted drugs and biologics, one tactic for tolerance induction is to add a targeted therapeutic medication alongside OIT. **Table 1** summarizes biomarkers of tolerance and biologics that have preliminary evidence suggesting they may be useful for targeting of a specific biomarker. Ongoing and future clinical trials will be needed to elucidate how well the biologics, when used in combination with IT, actually influence a particular biomarker and tolerance overall.

**Table 1:** Biomarkers of tolerance in food allergy IT studies and potential corresponding IT adjuvants.

Biomarker	Baseline biomarker	Biomarkers during build-up and maintenance OIT	Potential IT adjuvant
Age	Younger age (22, 48) No age association (24, 49)		
Total IgE	Lower total IgE (24)		
Food-specific IgE	Lower psIgE level (18, 22, 24, 48) Lower msIgE level (50, 68) Lower esIgE level (23) No esIgE association (21)		Anti-IgE (69) Anti-IL4Rα (70) JAK inhibitors (71) Anti-IL-13 (72, 73)
Food component-specific IgE	Lower Ara h 1-specific IgE level (18, 21) Lower Ara h 2-specific IgE level (18, 21, 51) Lower Ara h 3-specific IgE level (21, 51) Lower Ara h 6-specific IgE level (21) Lower ovomucoid-specific IgE level (23) No ovomucoid-specific IgE association (21) No ovalbumin-specific IgE association (21, 23) No Ara h 1-specific IgE association (22) No Ara h 2-specific IgE association (22) No Ara h 3-specific IgE association (22) No Ara h 6-specific IgE association (22)		Anti-IgE (69) Anti-IL4Rα (70) JAK inhibitors (71) Anti-IL-13 (72, 73)
Linear epitope profile	Lower levels of peanut epitope-specific IgE (55)		
Food-specific IgG4:IgE ratio	Higher peanut-specific IgG4:IgE ratio (24)		
Basophil activation test	Lower basophil activation (9, 45, 46) No basophil activation association (21, 40, 43)	Lower basophil activation at end of build-up or during maintenance (43–45)	Anti-IgE (74) JAK inhibitors (71) BTK inhibitor (75, 76) Anti-IL-13 (77) Anti-TSLP (78)
Dendritic cells		Decreased DC secretion of IL-6 (8)	Anti-TSLP (79)
Food-specific T effector cells	Lower number of psTeff cells producing IL-4 and/or IL-13 (21) Lower expression of gene module defined by T cell activation and effector response (33) Lower frequency of peanut-specific Th1-conv cells (33) Lower frequency of Th17 cells (33) No association with psTh2 cells (33) No association with IL-10 producing psTeff (21) No association with IFN-γ producing psTeff (21)	Evidence of CD4+ T cell anergy in maintenance phase of OIT (33, 34) Transient increase in activated psTh2 cells (39) Transient increase in TGF-β producing psTh2 cells (39)	JAK inhibitors (80, 81) Anti-OX40 (82) Anti-TSLP (83)
T regulatory cells	No Treg cell association (21)		
T follicular helper cells		Tfh13 and Tfh2-like cell frequencies did NOT associate with tolerance (33, 39)	Anti-IgE (84) Anti-OX40 (85)

IT, immunotherapy ps, peanut-specific; ms, milk-specific; es, egg-specific; DC= dendritic cell; Teff, T effector cells (154+CD4+ cells after antigen stimulation); Treg, T regulatory; Tfh, T follicular helper.

The most well-studied example of this is OIT plus omalizumab. Studies have shown that adding omalizumab, thereby blocking IgE from binding to mast cells, has been helpful for either reducing adverse reactions during OIT up-dosing or for allowing expedited up-dosing (50, 86, 87). It is not clear that this approach leads to increased rates of tolerance, but additional trials are still underway (NCT03881696, NCT04045301). A broader approach of immune modulation or an approach targeted to another aspect of the allergic immune response may be required to improve rates of tolerance.

Additional OIT plus trials include the Adjuvant Treatment with abatacept to Promote Remission During Peanut Oral Immunotherapy (ATARI) trial (NCT04872218) and a phase 2a trial of dupilumab plus Palforzia (NCT03682770). Abatacept is a CTLA-4-immunoglobulin fusion protein that binds to CD80 and CD86 on antigen presenting cells thus preventing T cells from receiving the activating signal they need to respond to antigen presentation (88). Without this signal, the balance of T effector, regulatory, and helper cells could be restored and in studies of autoimmunity, abatacept has been shown to decrease T follicular helper cells and T regulatory cells (88). Dupilumab is an IL-4 receptor alpha antagonist and blocks signaling of both IL-4 and IL-13. In atopic individuals, dupilumab leads to a reduction in a number of biomarkers of type 2 inflammation, including serum and/or plasma levels of thymus and activation-regulated chemokine (TARC), eotaxin-3, periostin, and total IgE, which could provide an immune environment that is more conducive to the induction of tolerance (70).

Ongoing trials of biologics as monotherapy for food allergy are also paving the way for subsequent OIT plus studies and include a pilot of abrocitinib, a selective JAK inhibitor, for adults with peanut allergy (NCT05069831). A number of cytokines relevant to food allergy signal through JAK1 including TSLP, IL-4, IL-13, and IL-9 (80). JAK inhibitors have already been shown to modulate these cytokines in atopic dermatitis trials (81) and by blocking signaling of these cytokines abrocitinib has the potential to decrease Th2 induction, induce Tregs, inhibit IgE class-switching, and inhibit mast cell expansion.

Potential future candidates for OIT plus trials include BTK inhibitors, Anti-OX40 antibody, anti-IL-13 antibodies, anti-TSLP antibody, and other JAK inhibitors.

## Conclusions

Performing mechanistic studies in conjunction with food immunotherapy clinical trials has taught us about mechanisms of tolerance and has helped to identify biomarkers of tolerance. This information is guiding the development of current and future clinical trials. It is imperative that we continue to perform mechanistic assays to improve our understanding of tolerance if we want to move beyond inducing only desensitization and tolerance to only one bite of a food with immunotherapy.

## Author contributions

Draft manuscript preparation: CB, MF, and TL. All authors contributed to the article and approved the submitted version.

## Funding

The work of the authors was supported by the National Institute of Allergy and Infectious Diseases of the National Institutes of Health under Award Number UM1AI109565.

## Acknowledgments

The authors would like to acknowledge the investigators and their teams who performed the trials and the mechanistic assays. We would additionally like to acknowledge the patients and families who participated in the clinical trials discussed here. We thank the Immune Tolerance Network (ITN) for the unique opportunity to study mechanisms of tolerance across different clinical trials in atopy.

## Conflict of interest

The authors declare that the research was conducted in the absence of any commercial or financial relationships that could be construed as a potential conflict of interest.

## Publisher’s note

All claims expressed in this article are solely those of the authors and do not necessarily represent those of their affiliated organizations, or those of the publisher, the editors and the reviewers. Any product that may be evaluated in this article, or claim that may be made by its manufacturer, is not guaranteed or endorsed by the publisher.

## Author disclaimer

The content is solely the responsibility of the authors and does not necessarily represent the official views of the National Institutes of Health.
